# Multimodal Reconstruction Approaches in Diabetic Foot Ulcers

**DOI:** 10.7759/cureus.86013

**Published:** 2025-06-14

**Authors:** Vijaykharthik LK, Manimaran R, Koppolu Kanchana, BV Sreedevi

**Affiliations:** 1 General Surgery, Sree Balaji Medical College and Hospital, Chennai, IND; 2 Plastic Surgery, Sree Balaji Medical College and Hospital, Chennai, IND

**Keywords:** diabetic foot ulcer, plantar flap, reconstruction, split-thickness skin graft, sural flap

## Abstract

Introduction: Diabetic foot ulcers (DFUs) are a common and serious complication of diabetes mellitus, often leading to prolonged morbidity and limb amputation. Successful management involves early debridement, infection control, and appropriate soft tissue coverage.

Methods: The present study is a retrospective case series conducted from March 1 to August 31, 2024, involving seven patients with chronic DFUs at Sree Balaji Medical College and Hospital. Each case underwent individualized reconstruction strategies, including split-thickness skin grafts (STSGs), reverse sural artery flaps, medial plantar artery flaps, cross-leg flaps, and conservative healing, depending on wound location, depth, and vascular status.

Results: All patients demonstrated favorable outcomes in terms of graft/flap viability and wound healing. Functional recovery was satisfactory in each case. No major complications or recurrences were observed during follow-up.

Conclusion: Early and tailored multimodal reconstructive approaches yield positive outcomes in DFUs. Strategic planning based on anatomical site and biomechanical load is crucial for effective limb salvage.

## Introduction

Diabetic foot ulcers (DFUs) are a significant complication of diabetes mellitus, affecting up to 25% of diabetic patients during their lifetime and often leading to hospitalization, morbidity, and even amputation if inadequately managed [1]. Their pathogenesis is multifactorial, involving peripheral neuropathy [2], peripheral arterial disease (PAD) [3], and impaired wound healing due to hyperglycemia-induced immune dysfunction [4,5].

Despite advancements in wound care, optimal surgical reconstruction remains complex and highly individualized. Effective management requires a multidisciplinary approach that includes strict glycemic control, appropriate antibiotic therapy, surgical debridement, and vascular optimization, all guided by evidence-based protocols [2,6]. Wound bed preparation may be enhanced by negative-pressure wound therapy to promote granulation and reduce bioburden [7]. Once the wound is adequately prepared, selecting an appropriate reconstructive method based on ulcer characteristics, such as location, size, and depth, is critical for achieving durable closure and restoring function. Split‐thickness skin grafts (STSGs) are typically used for superficial, well‐granulated defects [8,9]; reverse sural artery flaps are employed for posterior heel and lateral foot coverage [10,11]; medial plantar artery flaps are indicated for weight‐bearing plantar wounds [12,13]; and cross‐leg flaps are reserved for extensive or complex dorsolateral defects when local options are exhausted [5].

This case series presents seven patients with DFUs across diverse anatomical sites, each managed with a tailored reconstructive technique. By illustrating outcomes in various clinical scenarios, we demonstrate the effectiveness of a staged, individualized approach to limb salvage in the management of DFUs.

## Materials and methods

This retrospective case series was conducted at the Departments of General and Plastic Surgery, Sree Balaji Medical College and Hospital, Chennai, India. The study period was from March 1, 2024, to August 31, 2024. A total of seven patients with chronic DFUs were included. Inclusion criteria comprised adult patients with type 2 diabetes mellitus and chronic non-healing ulcers of the foot lasting >4 weeks, requiring surgical reconstruction. Patients with acute limb ischemia, active osteomyelitis, or systemic sepsis at presentation were excluded. All patients underwent detailed preoperative evaluation, including vascular assessment through peripheral Doppler studies, glycemic status monitoring, and nutritional optimization. Wound cultures were obtained, and antibiotics were administered based on sensitivity reports. Initial management involved serial sharp debridement, infection control, and use of negative-pressure wound therapy (also known as vacuum-assisted closure, VAC) where indicated. Once a healthy granulation bed was established, reconstruction was performed based on the ulcer site and biomechanical demand.

Reconstruction strategies included STSGs for superficial, granulating ulcers; medial plantar artery flaps for weight-bearing plantar defects; reverse sural artery flaps for heel and lateral foot ulcers; cross-leg flaps for large lateral defects lacking adjacent healthy tissue; and conservative management for small superficial ulcers. Postoperative care involved dressing changes, pressure offloading, and glycemic control. Patients were followed up for at least six weeks to assess wound healing, flap/graft survival, and functional outcomes.

## Results

Case 1: left post-forefoot amputation with STSG

**Figure 1:**
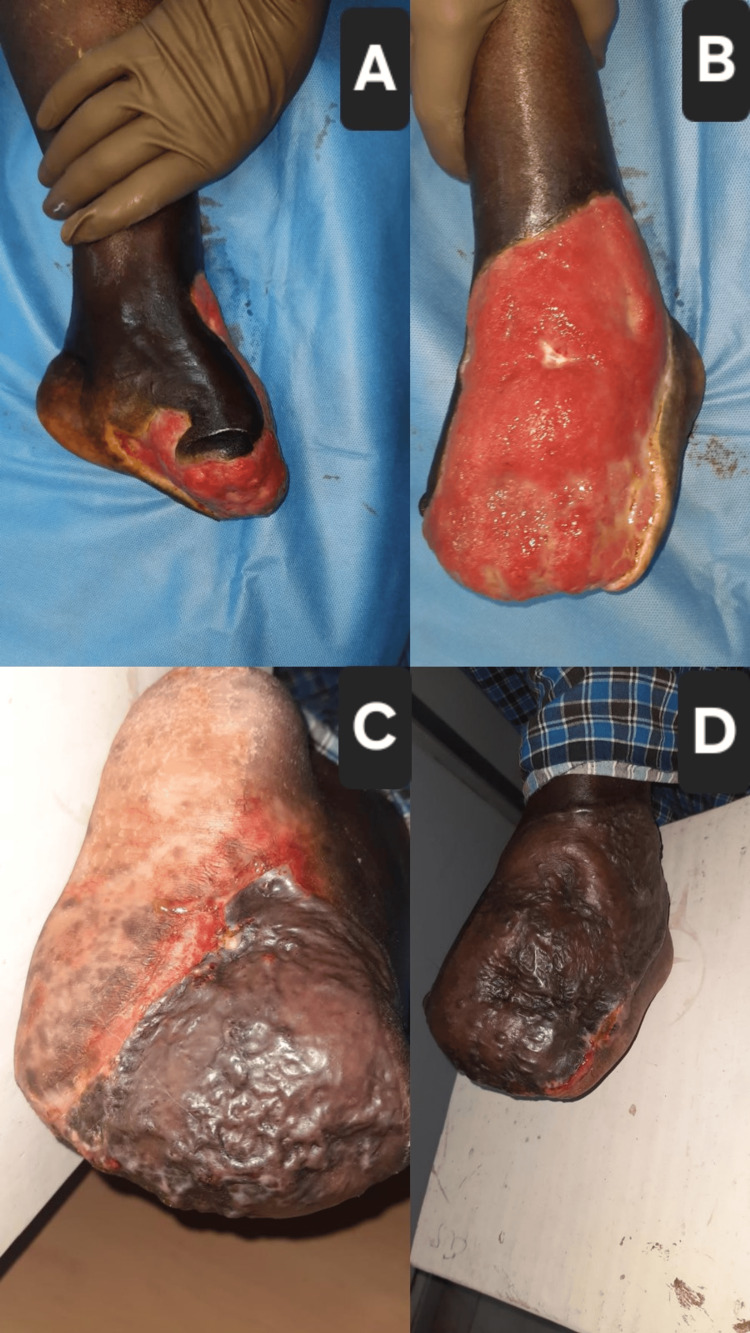
Case 1: Progression of left post-forefoot amputation stump reconstruction (A) Pre-grafting appearance of the partially amputated forefoot stump showing necrotic margins and devitalized tissue; (B) after serial debridement and VAC therapy, showing a well-vascularized granulation tissue covering the defect; (C) intraoperative view immediately following placement of an STSG harvested from the thigh; (D) four-week postoperative follow-up demonstrating 100% graft take with stable, durable coverage and no signs of infection. VAC: vacuum-assisted closure; STSG: split‐thickness skin graft

A 64-year-old male with a 20-year history of type 2 diabetes mellitus and hypertension presented with a necrotic ulcer involving the forefoot of the left foot, resulting in a partial forefoot amputation. The patient had blackish discoloration of the toes for over a week prior to presentation, with surrounding cellulitis and foul-smelling discharge. On inspection, the wound measured approximately 9×7 cm, with healthy red granulation tissue visible over the amputation stump and surrounding devitalized tissue (Figures 1A, 1B). Peripheral pulses were palpable. He underwent serial surgical debridements to remove all necrotic tissue and prepare the wound bed. Once the granulation tissue was mature and the infection controlled, the defect was covered with an STSG harvested from the right thigh (Figures 1C, 1D). The graft uptake was excellent, and the patient resumed partial weight-bearing by the fourth postoperative week.

Case 2: post-burn left plantar wound treated with STSG

**Figure 2:**
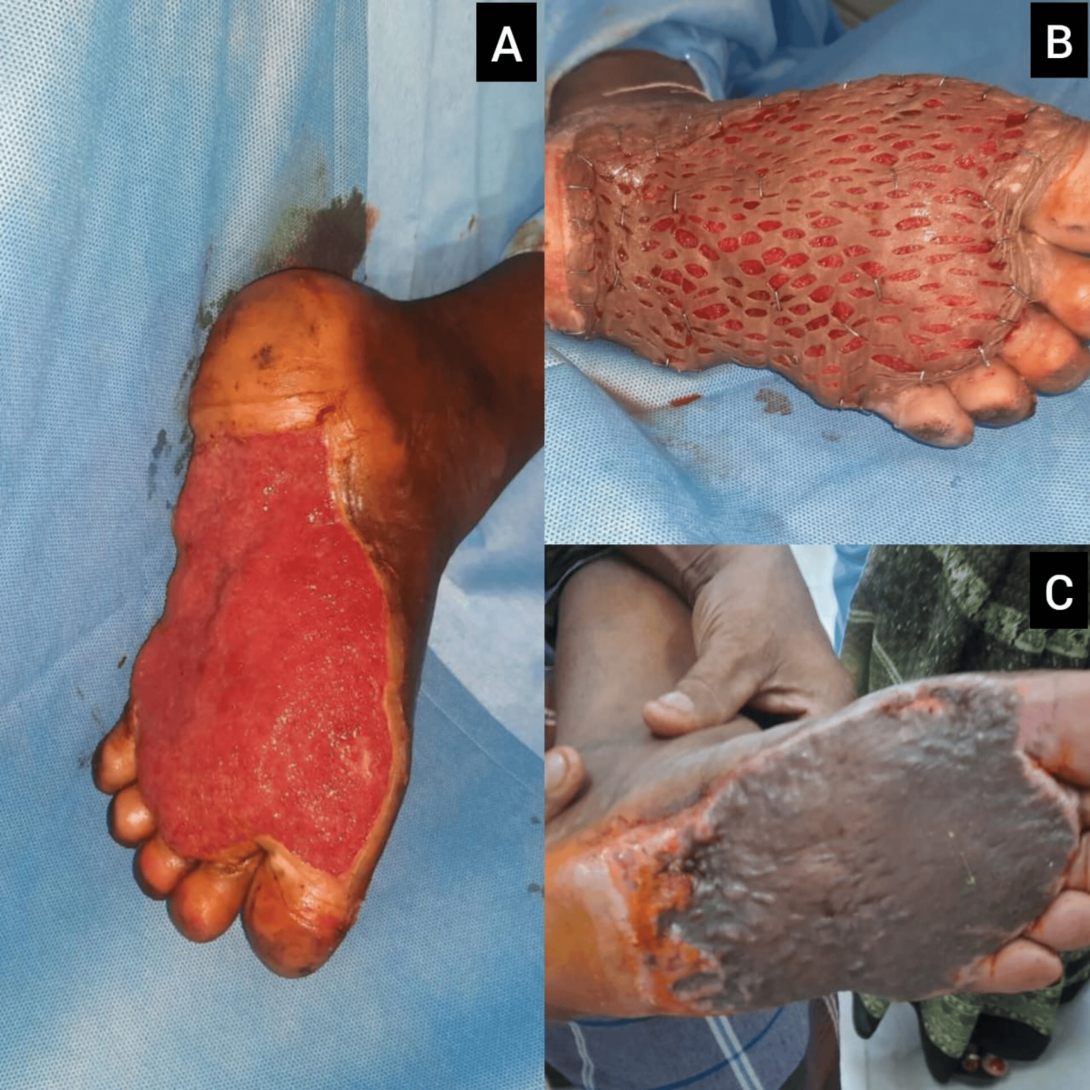
Case 2: Reconstruction of post-burn left plantar ulcer with STSG (A) Preoperative view of a large post-burn ulcer involving the plantar aspect of the left foot, with healthy granulation tissue extending from the heel to the forefoot; (B) intraoperative image following placement of an STSG harvested from the contralateral thigh; (C) three-week postoperative image showing near-complete graft take and well-epithelialized plantar surface, supported by offloading footwear. STSG: split‐thickness skin graft

A 59-year-old female diabetic patient presented with a large post-burn raw area involving the plantar aspect of the left foot, sustained from accidental exposure to hot cement flooring. The ulcer was extensive, measuring approximately 15×7 cm, extending from the heel to the base of the toes, with bright red granulation tissue and healthy margins (Figure 2A). There were no signs of cellulitis or active infection. Peripheral pulses were palpable. Following multiple sessions of wound debridement, the area was grafted with an STSG harvested from the contralateral thigh (Figure 2B). The graft take was near-complete (Figure 2C), and the patient was mobilized using pressure offloading footwear.

Case 3: medial plantar ulcer managed with medial plantar artery flap and STSG

**Figure 3:**
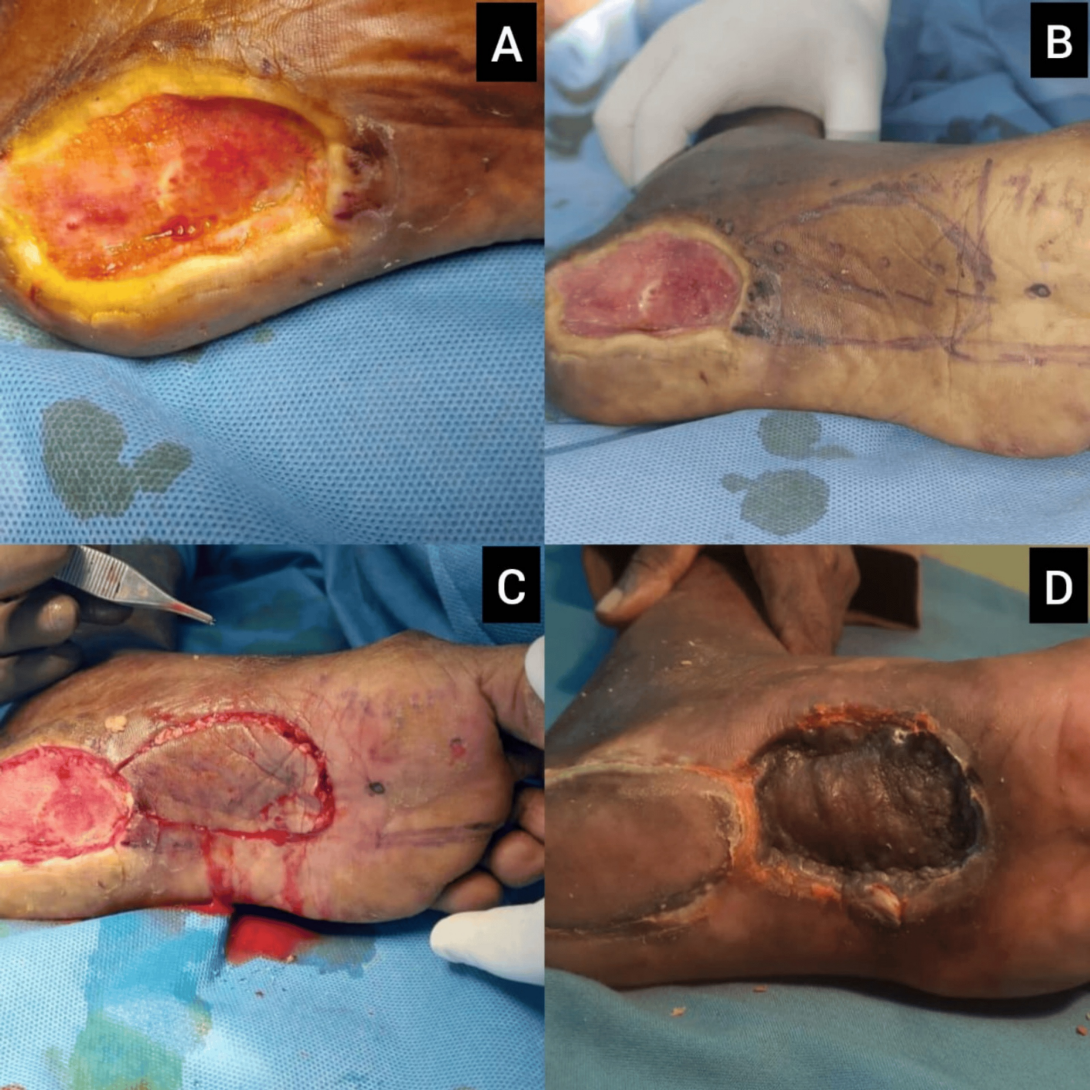
Case 3: Coverage of chronic medial plantar ulcer using medial plantar artery flap and STSG (A) Initial presentation of a chronic medial plantar ulcer with slough and maceration over a pressure-bearing zone; (B) post-debridement wound showing a clean bed prepared for flap coverage; (C) intraoperative view of the medial plantar artery flap rotated into the defect, with donor site covered using an STSG; (D) follow-up at three weeks showing excellent flap integration and stable wound closure with no breakdown. STSG: split-thickness skin graft

A 67-year-old male presented with a chronic non-healing ulcer located on the medial plantar aspect of the left foot. The ulcer measured approximately 4×3 cm, with a yellowish slough base and surrounding maceration (Figures 3A, 3B). The area was mildly tender, with no systemic signs of infection. Peripheral pulses were intact. The patient underwent repeated wound debridements to remove slough and biofilm. Given the location over a pressure-bearing zone, a medial plantar artery flap was raised and rotated to cover the defect (Figures 3C, 3D). The donor area was closed with an STSG, achieving good coverage. The patient was advised non-weight-bearing mobilization initially, followed by gradual rehabilitation.

Case 4: right heel ulcer treated with cross-leg reverse sural artery flap

**Figure 4:**
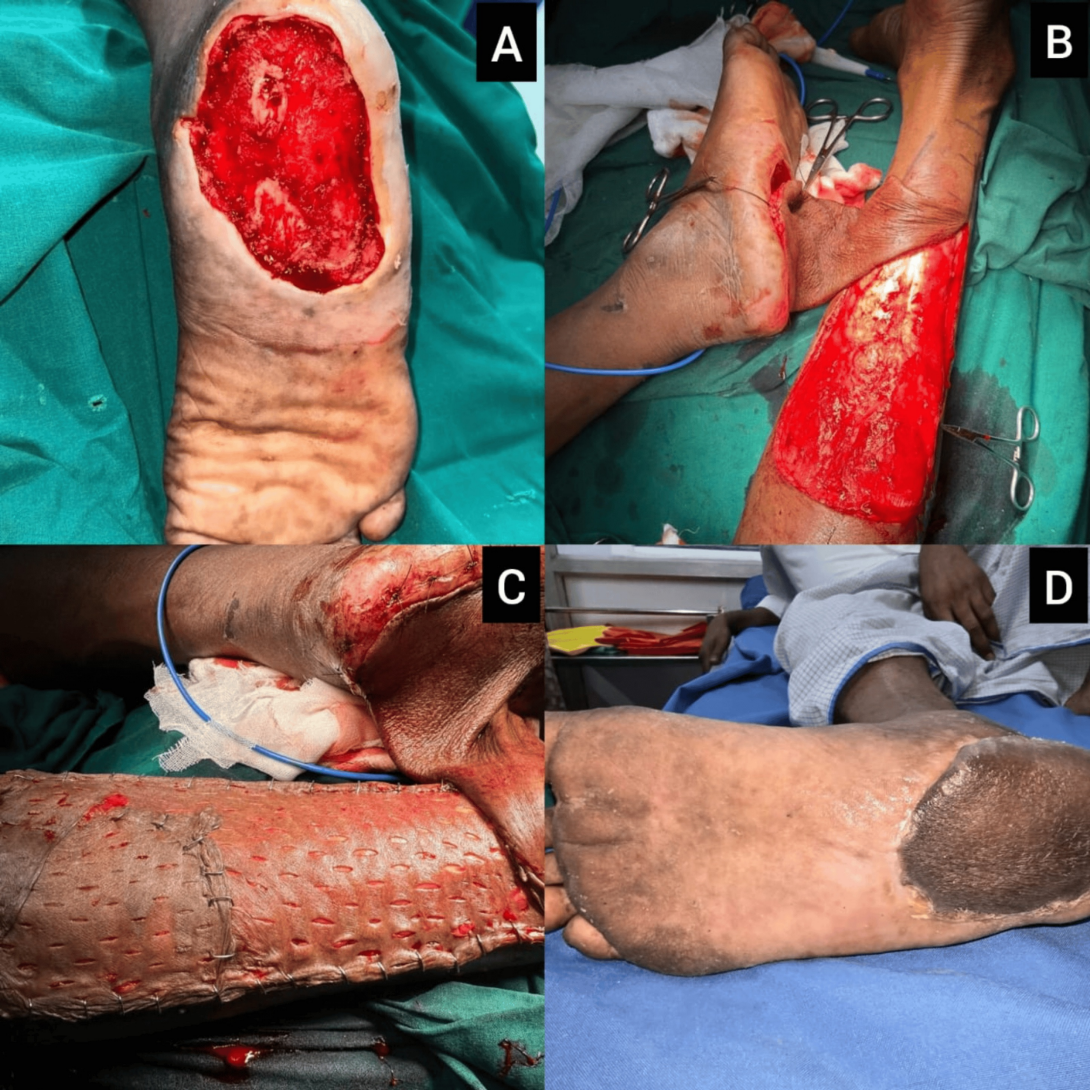
Case 4: Cross-leg reverse sural artery flap for right heel ulcer reconstruction (A) Preoperative view of a large right heel ulcer with exposed calcaneus and hemorrhagic base in a diabetic nephropathy patient; (B) after serial debridements, the wound demonstrates healthy granulation but remains too extensive for local closure; (C) intraoperative image of the cross-leg reverse sural artery flap transferred from the contralateral leg, secured over the defect; (D) postoperative result at six weeks showing full flap survival, healed donor site with STSG, and restoration of heel contour. STSG: split-thickness skin graft

A 58-year-old male with diabetic nephropathy presented with a large heel ulcer on the right foot that developed after prolonged immobilization. On examination, the wound was approximately 9×8 cm, exposing the calcaneus and underlying deep tissues, with irregular, hemorrhagic granulation at the base (Figure 4A). He underwent serial sharp debridement and dressing. Due to the size, depth, and lack of adjacent healthy tissue, a cross-leg reverse sural artery flap was planned. A cross-leg flap was raised from the contralateral leg and tunneled over to the defect. The donor site was covered with an STSG, and the flap showed excellent vascularity without signs of congestion or necrosis (Figures 4B, 4D). By postoperative week two, the flap and donor site had healed well, and the pedicle division was performed uneventfully at three weeks. The patient achieved protected weight-bearing ambulation by six weeks, with full epithelialization and no signs of ulcer recurrence or wound dehiscence. Functional recovery was assessed by the ability to ambulate independently with a walker, the absence of wound-related complications, and the return to preoperative levels of activity by eight weeks.

Case 5: left diabetic septic foot treated with VAC and STSG

**Figure 5:**
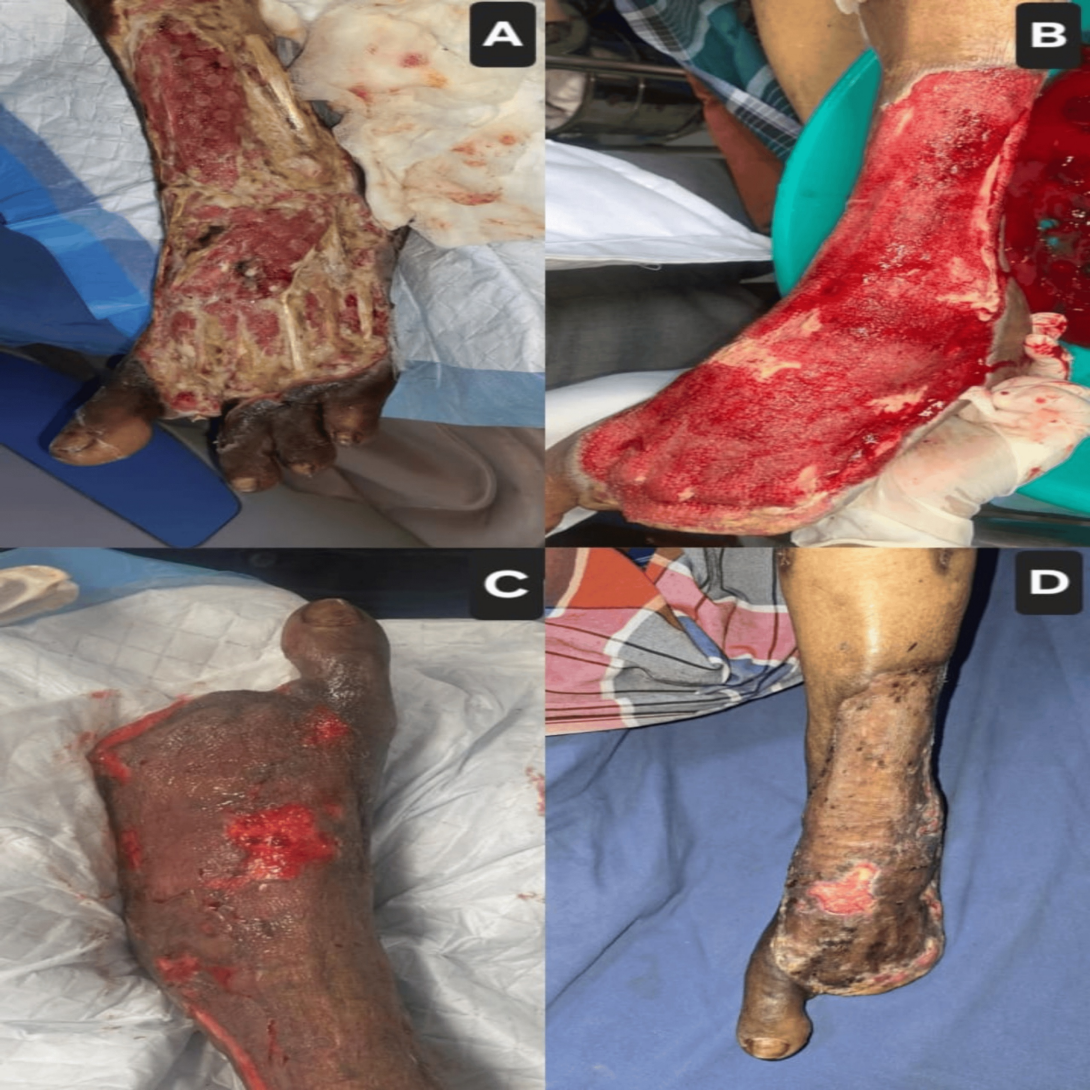
Case 5: VAC therapy and STSG in the management of diabetic septic foot (A) Preoperative view showing extensive circumferential necrosis and tissue loss involving the lower leg and foot with exposed tendons and devitalized tissue; (B) post-debridement wound bed following VAC therapy with well-formed granulation tissue; (C) intraoperative image after placement of an STSG; (D) three-week postoperative follow-up demonstrating healthy graft uptake and progressive epithelialization, with salvaged limb function. STSG: split-thickness skin graft

A 61-year-old female with longstanding type 2 diabetes mellitus presented with a septic foot and extensive tissue necrosis extending from the lower leg to the dorsum and plantar aspect of the foot. The wound was foul-smelling, with full-thickness skin loss exposing tendons and muscles, and patches of blackish discoloration indicative of dry gangrene (Figure 5A). Peripheral pulses were weakly palpable. Emergency radical debridement was performed to excise all necrotic and infected tissues. Due to the size, depth, and circumferential nature of the defect, VAC therapy was initiated and continued for 10 days. The VAC promoted rapid granulation tissue formation (Figure 5B), significantly reducing wound dimensions and preparing the site for definitive reconstruction. Upon achieving a healthy granulating bed, an STSG was harvested from the contralateral thigh and subsequently secured over the defect (Figure 5C). The graft was well taken with no evidence of infection, and the patient showed stable healing at the three-week follow-up with no need for major amputation (Figure 5D).

Case 6: right heel pad ulcer treated with medial plantar artery flap

**Figure 6:**
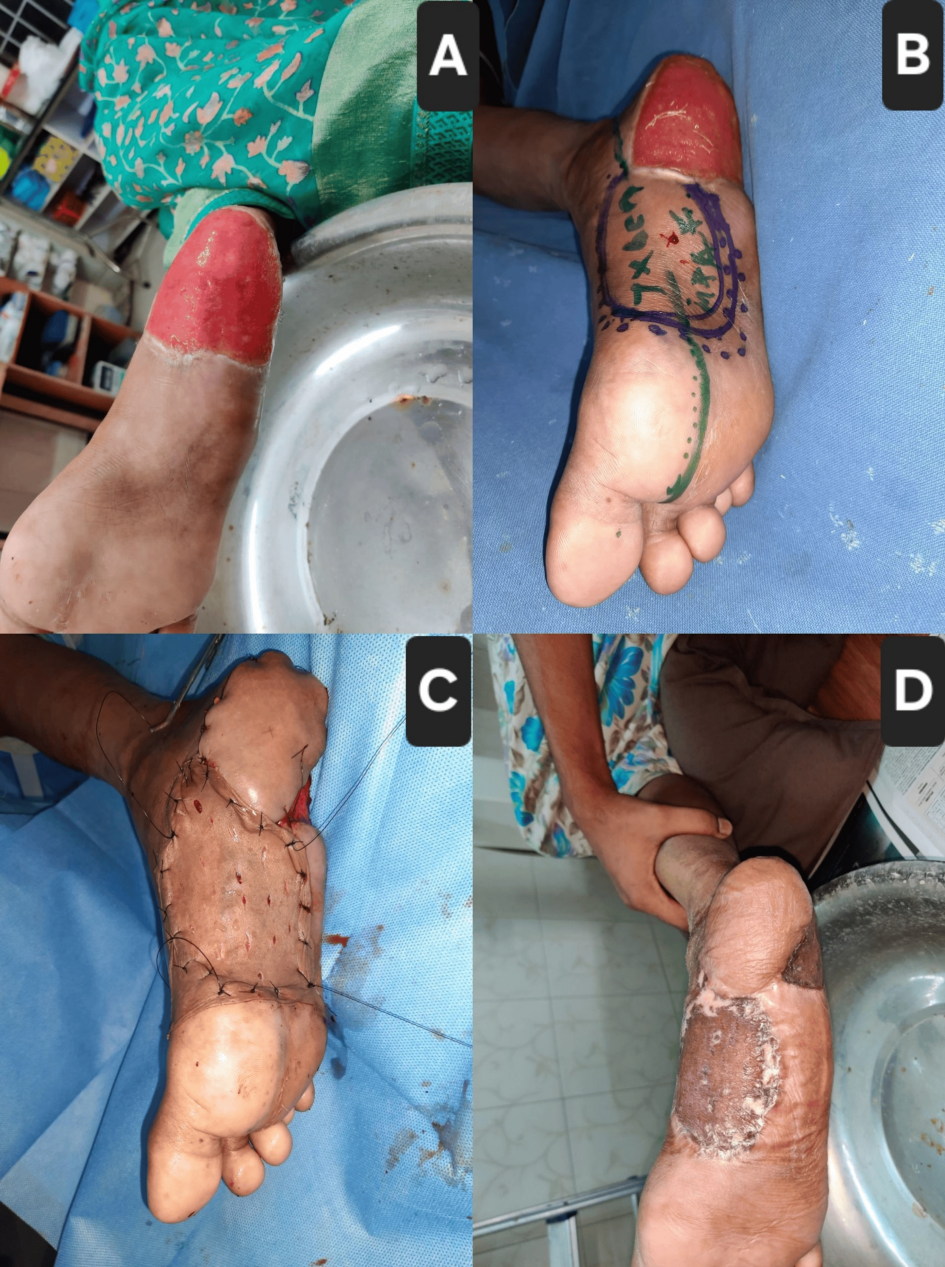
Case 6: Right heel pad ulcer reconstruction using medial plantar artery flap (A) Preoperative image showing a well-granulated distal plantar defect post transmetatarsal amputation; (B) flap design marked over the medial plantar region, indicating the flap axis and neurovascular pedicle; (C) intraoperative view after inset of the medial plantar artery flap, with donor site covered by STSG; (D) four-week postoperative outcome with stable wound coverage and excellent flap viability. STSG: split-thickness skin graft

A 56-year-old diabetic female with ischemic heart disease presented with a well-granulated distal plantar wound following transmetatarsal amputation of the right foot (Figure 6A). The defect measured approximately 6×6 cm, with exposed fibrofatty tissue and a healthy granulation bed. The surrounding skin was intact but discolored. Peripheral pulses were palpable. After repeated debridement and wound bed optimization, a medial plantar artery flap was harvested and transposed to cover the plantar defect (Figures 6B, 6C). Flap vascularity was satisfactory, and the patient began protected heel weight-bearing at four weeks postoperatively with excellent functional recovery.

Case 7: right foot plantar ulcer treated with debridement and secondary healing

**Figure 7:**
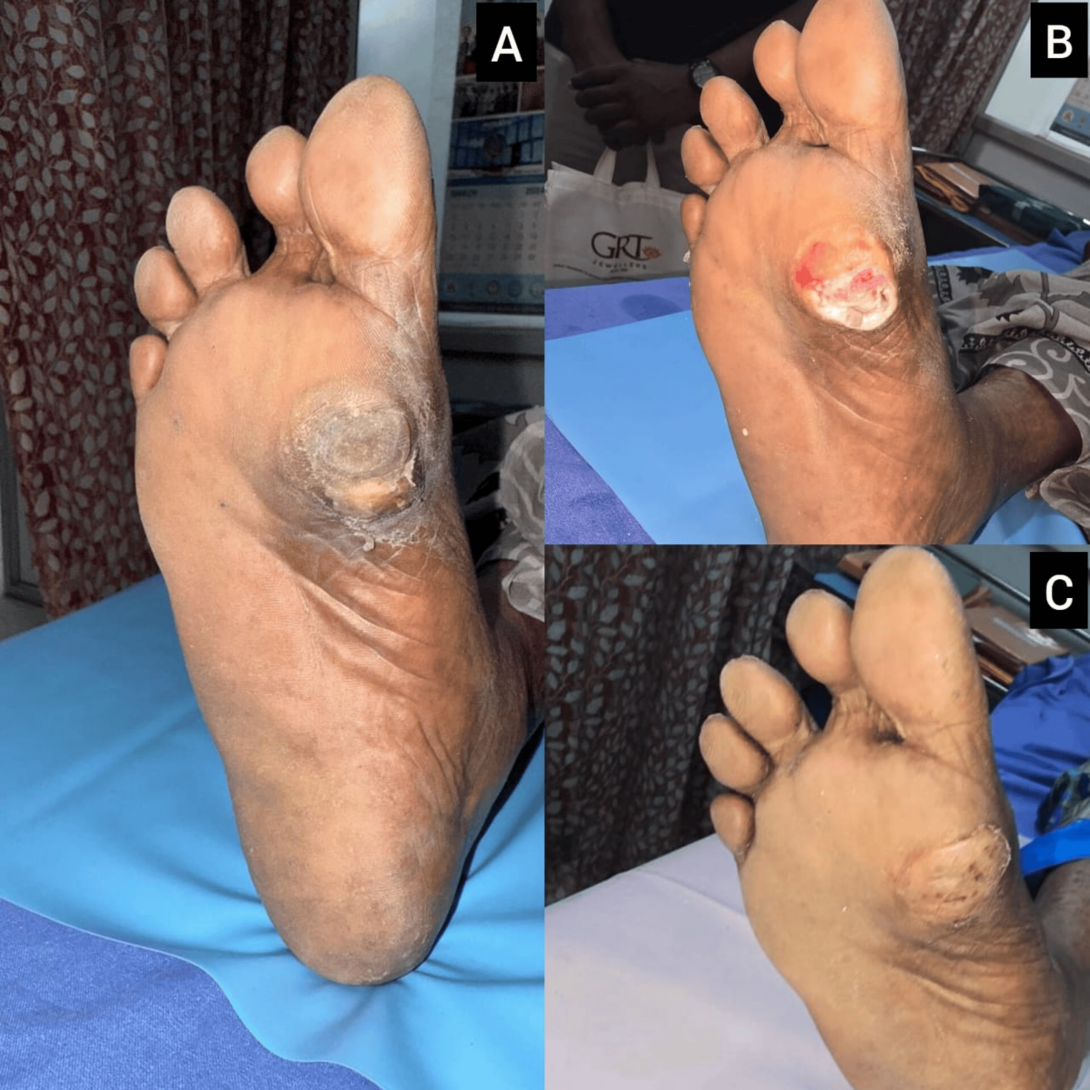
Case 7: Management of plantar ulcer with secondary intention healing (A) Initial presentation of a dry, circular ulcer over the second metatarsal head with peripheral callosity; (B) after sharp debridement, the wound shows a clean base with early granulation; (C) mid-treatment phase showing reduction in wound size and progressing epithelialization.

A 60-year-old female presented with a chronic plantar ulcer over the right forefoot, overlying the second metatarsal head. The lesion was circular, dry, and measured about 2.5×2.5 cm, with peripheral callosity and central crusting (Figure 7A). There were no signs of infection or discharge. Peripheral pulses were well felt. The patient underwent sharp debridement and daily saline dressings (Figures 7B, 7C). Offloading footwear was provided, and the ulcer showed progressive healing by secondary intention over four weeks. Full healing was achieved without the need for surgical intervention.

The time from initial presentation to complete wound healing ranged from three to six weeks in most cases following debridement and reconstruction. This case series presents seven patients with chronic plantar ulcers managed at our institution, each with varying underlying comorbidities, ulcer etiologies, and anatomical locations. All patients were diabetic, with some having additional risk factors such as hypertension, chronic kidney disease (CKD), or ischemic heart disease. The ulcers were localized predominantly over high-pressure weight-bearing areas of the foot, including the forefoot, heel, and mid-plantar regions. Reconstruction techniques were selected based on wound size, depth, vascularity, and patient-specific factors, ranging from conservative secondary intention healing to advanced flap coverage. Glycated hemoglobin (HbA1c) levels were recorded in five of the seven patients at presentation. Case 1 had an HbA1c of 7.1%, indicating moderately controlled diabetes. Case 2 had a well-controlled level of 6.8%. Case 3 showed suboptimal control with an HbA1c of 8.3%, while Case 4 had a level of 9.1%, reflecting poor glycemic control. Case 6, with ischemic heart disease, had an HbA1c of 7.4%. HbA1c values were not available for Cases 5 and 7 due to a lack of recent laboratory records and urgent surgical debridement before the routine workup, respectively. A summary of the demographic characteristics, ulcer location, surgical procedures performed, type of reconstruction, and clinical outcomes is provided in Table 1.

**Table 1:** Summary of cases undergoing multimodal reconstruction for diabetic foot ulcers T2DM: type 2 diabetes mellitus; CKD: chronic kidney disease; IHD: ischemic heart disease; PAD: peripheral arterial disease; STSG: split-thickness skin graft; VAC: vacuum-assisted closure

Case No.	Age/Sex	Comorbidities	Ulcer Location	Procedure Performed	Reconstruction Type	Outcome
1	64/M	T2DM, hypertension	Left plantar forefoot (post-forefoot amputation)	Serial debridement, STSG	Split-thickness skin graft	Complete healing in 4 weeks
2	59/F	T2DM	Left plantar (post-burn)	Serial debridement, STSG	Split-thickness skin graft	Graft uptake complete; healed in 3 weeks
3	67/M	T2DM, hypertension	Left medial plantar region	Serial debridement, medial plantar artery flap + STSG	Local flap + skin graft	Flap viable; full healing in 5 weeks
4	58/M	T2DM, CKD stage II	Right heel	Serial debridement, cross-leg reverse sural flap + STSG	Cross-leg pedicled flap	Flap survived; healed in 6 weeks
5	61/F	T2DM	Left foot (dorsum + plantar)	Debridement, VAC therapy, STSG	VAC + skin graft	Excellent healing; graft viable
6	56/M	T2DM, IHD	Right heel pad	Serial debridement, medial plantar artery flap	Local sensate flap	Healed well; resumed weight-bearing
7	60/F	T2DM	Right plantar metatarsal head	Serial debridement, secondary healing	Conservative management	Healed by secondary intention in 4 weeks

## Discussion

DFUs are a serious complication of diabetes mellitus, associated with significant morbidity and limb loss. These wounds result from a combination of peripheral neuropathy, impaired immunity, and microvascular compromise [1]. The chronicity of these ulcers demands a multidisciplinary and staged approach, incorporating debridement, infection control, wound bed preparation, and timely reconstruction [2].

Our case series highlights the practical application of evidence-based diabetic wound care. The classification system developed by Armstrong et al., which takes into account depth, infection, and ischemia, was instrumental in our decision-making process for staging reconstruction [3]. The underlying pathophysiology, marked by impaired angiogenesis and dysfunctional fibroblasts, explains the delay in healing and necessitates surgical intervention in most cases [4,5]. Guidelines from the International Working Group on the Diabetic Foot (IWGDF) recommend an aggressive, multidisciplinary strategy tailored to patient comorbidities and ulcer characteristics [6]. Many of our patients presented with PAD, CKD, or neuropathy, which are known predictors of delayed healing [7,8]. These systemic conditions complicate both wound progression and surgical planning.

The economic burden of DFUs is substantial. Rice et al. highlighted that the cost of treating chronic diabetic foot wounds can exceed that of many common cancers, which further underscores the need for efficient, structured management [9]. VAC therapy has emerged as a crucial adjunct for wound bed preparation. It helps decrease local edema, reduces bacterial load, and stimulates the formation of granulation tissue [15]. In our series, VAC therapy was used in three patients prior to skin grafting or flap coverage. A lesser-discussed but vital aspect of diabetic wound care is the modulation of inflammation. Ennis et al. emphasized that chronic wounds in diabetics remain stuck in a persistent inflammatory phase and that resolution of this inflammation is essential for effective healing [12]. While our series did not involve advanced biological therapies, the principle of restoring an optimal inflammatory environment was achieved through serial debridement and targeted antimicrobial management, key steps prior to any reconstructive procedure, as shown in Table 2.

**Table 2:** Clinical strategies applied in the case series with literature support VAC: vacuum-assisted closure; STSG: split-thickness skin graft; PAD: peripheral arterial disease; CKD: chronic kidney disease; IHD: ischemic heart disease

Strategy	Clinical Application in This Series	Supporting References
Wound Classification	Used for planning reconstruction based on depth, infection, and ischemia	[2], [3]
Serial Surgical Debridement	Performed in all cases as initial wound bed preparation	[2], [4], [5]
VAC Therapy	Used in three patients to promote granulation prior to grafting	[10], [14]
STSG	Used in five patients for shallow, granulating ulcers	[1], [2], [6], [17]
Reverse Sural Artery Flap	Utilized in two heel ulcers with deep exposure	[6], [11], [13]
Medial Plantar Artery Flap	Used in two plantar and heel defects requiring durable, sensate coverage	[12], [13]
Cross-leg Flap	Applied in one case with a large lateral midfoot defect and no local options	[5], [13]
Nutritional Support	Tailored immunonutrition was initiated in all patients preoperatively	[17]
Functional Outcome Assessment	Postoperative recovery was evaluated per standard definitions and follow-up tools	[16], [17]
Comorbidity Management	PAD, CKD, neuropathy, and IHD are managed medically during and post-treatment	[6], [7], [17]

STSGs were used effectively in five patients with clean, granulating wounds. Zhou et al. and others have affirmed the role of STSG in managing superficial or planar foot defects, especially after VAC therapy [1,2]. Flap reconstruction, however, is essential for durable coverage in weight-bearing and structurally complex areas. Reverse sural artery flaps were used in two cases for heel coverage with good outcomes, consistent with the recommendations of Schaper et al. and others [6,11]. In one patient with a large lateral foot defect and no local tissue available, a cross-leg flap was performed, a technique that remains relevant for limb salvage despite its logistical demands [5,13]. Medial plantar artery flaps were chosen in two cases involving plantar and heel ulcers. These flaps are sensate, durable, and biomechanically suited for weight-bearing zones [17]. Their use is supported by long-term studies showing good functional outcomes and minimal donor site morbidity [12,13]. Selecting the reconstruction method based on biomechanical demand and ulcer location, as Frykberg et al. advocate, is essential in optimizing limb salvage outcomes [18], as shown in Table 3.

**Table 3:** Correlation of reconstruction technique with anatomical site and biomechanical demand STSG: split-thickness skin graft; IHD: ischemic heart disease; VAC: vacuum-assisted closure

Ulcer Site	Technique Used	Rationale	References
Plantar Forefoot (Post-amp)	STSG	Non-weight-bearing stump, well-granulated	[1], [2]
Heel (Post-burn, Posterior)	STSG/reverse sural flap	Moderate to deep wounds; durable coverage required	[6], [11]
Medial Plantar Region	Medial plantar artery flap	Sensate, stable flap for weight-bearing areas	[12], [13]
Lateral Midfoot	Cross-leg flap + STSG	No local option; deep and complex defect	[5], [13]
Plantar Metatarsal Head	Conservative healing	Superficial ulcer managed by offloading and debridement	[6], [8]

Postoperative outcomes in our series were favorable across all seven cases, with complete healing and no recurrence. Our follow-up incorporated standardized evaluation metrics, guided by definitions from Lazarus et al., ensuring objective assessment of graft or flap success [16]. Nutritional optimization was undertaken for each patient, following recommendations by Chow and Barbul on the role of immunonutrition in tissue repair [17]. Our results support the structured approach endorsed by Frykberg et al., combining surgical precision, systemic optimization, and patient-centered planning [18]. While we did not encounter osteomyelitis, awareness and vigilance remain important. Grayson et al. and Alvaro-Afonso et al. have shown that early recognition of bone involvement improves surgical outcomes, a concept we applied by thoroughly evaluating all ulcers before reconstruction [19,20], as shown in Table 4.

**Table 4:** Healing outcomes and evidence alignment VAC: vacuum-assisted closure

Outcome	Cases Observed	Related Evidence From Literature	References
Complete healing in <6 weeks	All 7 cases	Supports early surgical reconstruction and VAC use	[2], [10], [18]
No flap/graft failure	6 out of 6 cases	Flap viability associated with patient selection and prep	[6], [11], [13]
No recurrence at follow-up	All cases	Matches criteria for successful limb salvage	[3], [16], [18]
No infection or osteomyelitis	All cases	Effective debridement and wound control critical	[5], [19], [20]

While advanced modalities such as artificial dermal matrices and free tissue transfer are valuable in selected DFU cases, they were not employed in this series due to resource availability and patient comorbidities.

This study has several limitations. Firstly, it is an observational case series with a small sample size (n = 7), which limits both statistical power and generalizability. Secondly, the follow-up duration was relatively short (ranging from six to eight weeks), which prevented the long-term assessment of recurrence and sustained functional outcomes. Thirdly, while patients demonstrated satisfactory clinical recovery, no standardized functional scoring systems, such as the Foot Function Index (FFI) or Lower Extremity Functional Scale (LEFS), were formally applied postoperatively. The absence of both preoperative and postoperative scores limits objective quantification of functional improvement. Nonetheless, this study provides valuable real-world insights into the feasibility and outcomes of multimodal diabetic foot reconstruction in resource-constrained settings.

## Conclusions

This case series demonstrates that a multimodal, individualized surgical approach, incorporating debridement, negative-pressure wound therapy, and anatomically appropriate flap or graft coverage, can achieve favorable outcomes in complex DFUs. Despite the small sample size and lack of formal functional scoring, the results support the effectiveness of protocol-driven, interdisciplinary management in promoting limb salvage and functional recovery in resource-limited settings.
